# Genetic Studies of Vibrio cholerae in South West Cameroon—A Phylogenetic Analysis of Isolates from the 2010-2011 Epidemic

**DOI:** 10.1371/currents.outbreaks.13b4e5e36a5c0831a1663fbdb5713fe9

**Published:** 2016-08-12

**Authors:** Moise C. Ngwa, Thomas Masalla, Seraphine Esemu, Foche Francis Fumoloh, Ian Kracalik, Eleonora Cella, Meer Taifur Alam, Jane-Francis Akoachere, Song Liang, Marco Salemi, J. Glenn Morris, Afsar Ali, Lucy M. Ndip

**Affiliations:** Emerging Pathogens Institute, University of Florida, Gainesville, Florida, USA; Department of Environmental and Global Health, College of Public Health and Health Professions, University of Florida, Gainesville, Florida, USA; Laboratory for Emerging Infectious Diseases, University of Buea, Buea, South West Region, Cameroon; Laboratory for Emerging Infectious Diseases, University of Buea, Buea, South West Region, Cameroon; Laboratory for Emerging Infectious Diseases, University of Buea, Buea, South West Region, Cameroon; Emerging Pathogens Institute, University of Florida, Gainesville, Florida, USA; Emerging Pathogens Institute, University of Florida, Gainesville, Florida, USA; Laboratory for Emerging Infectious Diseases, University of Buea, Buea, South West Region, Cameroon; Emerging Pathogens Institute, University of Florida, Gainesville, Florida, USA; Department of Environmental and Global Health, College of Public Health and Health Professions, University of Florida, Gainesville, Florida, USA; Emerging Pathogens Institute, University of Florida, Gainesville, Florida, USA; Emerging Pathogens Institute, University of Florida, Gainesville, Florida, USA; Department of Medicine, College of Medicine, University of Florida, Gainesville, Florida, USA; Emerging Pathogens Institute, University of Florida, Gainesville, Florida, USA; Department of Environmental and Global Health, College of Public Health and Health Professions, University of Florida, Gainesville, Florida, USA; Laboratory for Emerging Infectious Diseases, University of Buea, Buea, South West Region, Cameroon; Department of Microbiology, University of Buea, Buea, South West Region, Cameroon

## Abstract

**Introduction**: During the cholera outbreak from 2010 to 2011 in Cameroon, 33,192 cases with 1,440 deaths (case fatality ratio 4.34%) were reported to the World Health Organization. Of these, the South West Region reported 3,120 clinical cases. This region is in the Equatorial Monsoon climatic subzone of Cameroon, close to the coast, raising questions as to whether cases were linked with development of environmental reservoirs.

**Methods**: In an investigation conducted by the Laboratory for Emerging Infectious Diseases, University of Buea, toxigenic *V. cholerae* O1 were isolated from diarrheal stool samples from 18 patients, with ages ranging from <3 to 70 years. Coordinates for clinical centers at which cases were identified were obtained using a handheld GPS, and were mapped using ArcGIS. Antibiotic susceptibility testing was performed using the Kirby ‘Bauer agar disc diffusion method. The full genomes of these strains were sequenced with the Illumina MiSeq platform. De novo assembly of cholera genomes and multiple sequence alignment were carried out using the bioinformatics pipeline developed in the Emerging Pathogens Institute laboratory at the University of Florida.

**Results/Discussion**: Genetic comparisons showed that isolates were closely related, with pairwise p-distances ranging from 2.25 to 14.52 10-5 nt substitutions per site, and no statistically significant correlation between the pairwise genetic distances and the geographic distances among sampling locations. Indeed, the phylogeny of the Cameroonian strains displays the typical star-like topology and intermixing of strains from different locations that are characteristic of an exponential outbreak localized around a relatively restricted area with occasional spillover to other parts of the country, likely mediated by direct human contact and human movement. Findings highlight the utility of whole genome sequencing and phylogenetic analysis in understanding transmission patterns at the local level.

## Introduction**

Between 2010 and 2011, Cameroon recorded its worst cholera outbreak since1971, when the disease was first reported in the country. In the 2010-2011 epidemic, 33,192 cholera cases with 1,440 deaths (case fatality ratio 4.34%) were reported to the World Health Organization.^1^^,^ 2 In the southern plateau of Cameroon, the South West Region was one of the heavily affected regions during the 2010/11 outbreak. This region is in the Equatorial Monsoon^3^ climatic sub zone of Cameroon, close to the coast, raising questions as to whether transmission was linked with the development of environmental reservoirs or human movement. This is a key question in developing cholera intervention strategies; it is also a question which has generated considerable controversy, with at least one group hypothesizing that cholera transmission in Africa is solely a function of human movement,^4^^,^ 5 without the environmental reservoirs as reported in Asia and other parts of the world. Further, given the magnitude of the outbreak, another key question was whether or not the outbreak’s causative isolate was resistant to the prevailing antibiotics. We sought to address these questions for the South West Region-Cameroon, making use of isolates and whole genome sequence data from the 2010/11 cholera epidemic.

## The Study

While clinical cases are incomplete for 2010, 2011 case data obtained from the ministry of public health of Cameroon showed evidence of three disease peaks, which did not correlate with seasonal patterns of rainfall (Figure 1); very few cases were recorded in 2012 while cases have not been identified in subsequent years. Toxigenic *V. cholerae* O1 altered biotype Ogawa was obtained from 18 patients (12% of rice-water stool samples collected) who were living in diverse geographic locations ranging from estuarine environmental sites along the coastline and inland sites, including Mundemba (Figure 2A). The ages of the patients ranged from <3 to 70 years. Samples were collected from October 2010 through to June 2011, and sampling sites included all health facilities (clinics, hospitals, and cholera treatment centers) reporting at least one case of cholera. Global positioning system (GPS) coordinates of each clinical sampling site were obtained using GARMIN eTrex 30 handheld GPS and maps were produced using ArcGIS 10.2.

**Total number of weekly cholera cases in the South West Region of Cameroon in 2010 and 2011.**
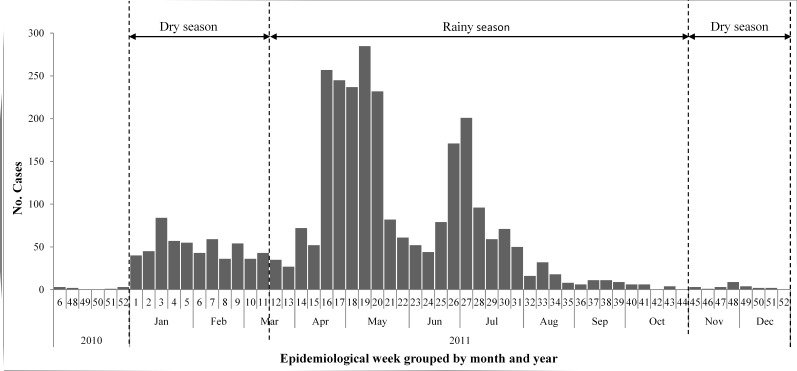
This region is in the Equatorial Monsoon climate sub zone of Cameroon. Cholera exhibits a trimodal transmission pattern with peaks in the dry (week 3) and rainy (weeks 19 and 27) seasons. Cholera clinical data was obtained from the ministry of public health of Cameroon.

**Geographic distribution of sampled V. cholerae strains in South West Region-Cameroon.**
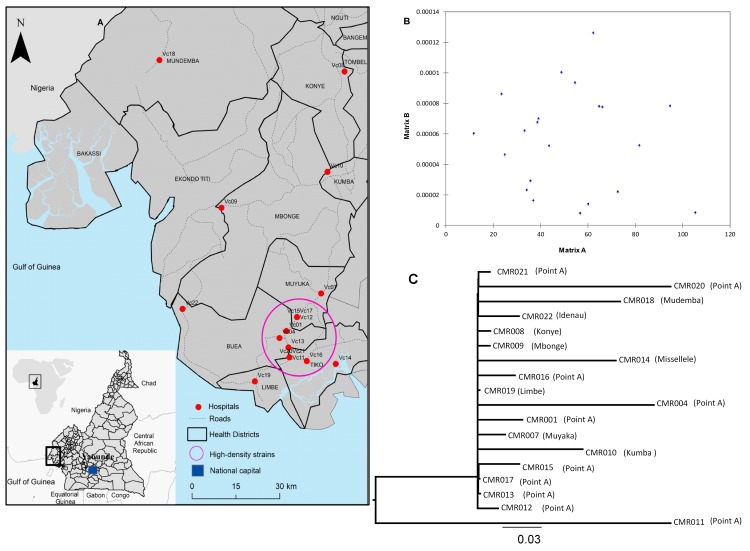
Panel A: red dots indicate the sample location of each strain in South West-Cameroon; the purple circle includes strains from the high-density populated area around the capital that likely harbored the origin of the outbreak. Panel B: pairwise genetic distances (y-axis) and geographic distances (in Km) among sampling locations (x-axis) were plotted to investigate specific migration trends of *V. cholerae* from the epicenter of the outbreak (purple circle) to the periphery. The two-tailed Mantel test failed to find any statistical correlation between the matrices of pair-wise genetic and geographic distances (p=0.74), indicating that strains genetic divergence does not increase with sampling locations progressively more distant and suggesting a homogeneous outbreak localized around a center, with occasional spillover in distant areas. Panel C: Maximum likelihood phylogenetic tree of full genome cholera strains from South West Region-Cameroon. The sampling location of each strain is given in parenthesis; ‘Point A’ represents areas enclosed within the purple circle in the map.

Fecal samples from suspect cases were analyzed in the Laboratory for Emerging Infectious Diseases, University of Buea, Cameroon. After initial isolation on thiosulfate citrate bile salts sucrose (TCBS) agar and passage on brain heart infusion agar (BHIA), oxidase-positive isolates from BHIA were presumptively identified as *V. cholerae* O1 by slide agglutination assay using polyvalent antiserums specific to *V. cholerae* O1 and O139. Polyvalent antiserum positive *V. cholerae* O1 isolates were differentiated to sub-serotypes by testing against monovalent antiserum (Ogawa, Inaba, and Hikojima). Each *V. cholerae* O1 isolate was further characterized using a panel of PCR and mismatch amplification mutation assay (MAMA) PCR primers targeting *V. cholerae* species specific and virulence specific genes, including *ctxA, ctxB* and *tcpA* genes, as described previously.^6^ Furthermore, we PCR amplified the entire *ctxB* gene from each Cameroonian isolate and the PCR amplicon was sequenced in the Interdisciplinary Center for Biotechnology Research (ICBR) at University of Florida. Sequence comparison showed that all 18 isolates have identical *ctxB* gene which aligned (100%) to the *ctxB7* 7 sequence of *V. cholerae* classical strain, O395, but differed from *ctxB3* sequence of N16961, an El Tor *V. cholerae* strain.^8 ^Our results confirm that the South West Region-Cameroonian isolates are *V. cholerae* strains carrying *ctxB7* gene mirroring classical *ctxB* in El Tor background as corroborated by the MAMA PCR.

Antibiotic susceptibility testing was performed using the Kirby ‘Bauer agar disc diffusion method following CLSI (Clinical and Laboratory Standards Institute)^9^ guidelines^9^^,^ 10 ^,^ 11 and disk were collected from Oxoid, Hampshire, UK. Susceptibility was determined to Amikacin (30 μg), Ampicillin (10 μg), Azithromycin (15 μg), Cefixime (five μg), Ceftazidime (30 μg), Ceftriaxone (30 μg), Cefotaxime (30 μg), Ciprofloxacin (5 μg), Chloramphenicol (30 μg), Doxycycline (30 μg), Gentamicin (120 μg), Tetracycline (30 μg), Nalidixic acid (30 μg) and Trimethoprim /Sulfamethoxazole (25 μg). Our results indicate that all the strains showed the same pattern of antibiotic susceptibility. They are resistant against Nalidixic acid and Trimethoprim/Sulfamethoxazole, but intermediate against Chloramphenicol and Ampicillin, and susceptible to the rest of the antibiotics used in the study (Supplemental Table S1)

The full genomes of all the 18 *V. cholerae* O1 isolates were sequenced with the Illumina MiSeq platform. De novo assembly of cholera genomes and multiple sequence alignment were carried out using our previously described bioinformatic pipeline.^12 ^There were a total of 2673 single nucleotide polymorphisms (**SNPs**) within the outbreak genomes after excluding repetitive and putative recombinant (identified with the program Gubbins^13^) regions. Sequence data have been deposited at DDBJ/ENA/GenBank under the accession XXXX00000000. The version described in this paper is version XXXX01000000 (Supplemental Table S2). Genetic comparisons showed that isolates were closely related, with pairwise p-distances ranging from 2.25 to 14.52 10-5 nt substitutions per site. Potential correlation between the pairwise genetic distances and geographic distances among strains sampling locations was investigated with a two-tailed Mantel test, using GPS coordinates of the sampling sites. No statistically significant correlation was found (p=0.74, Figure 2B). In addition, we evaluated whether there is a correlation between the genetic distance from the common ancestor (root of tree) and the geographical distance from the epicenter of the epidemic (i.e., the city of Buea). There was no association between the genetic distance from the root and geographic distance from the epicenter (Figure 3), which may reflect the impact of human mobility. Using phylogenetic analysis, genomic relatedness among the strains was further investigated. A maximum likelihood phylogenetic tree was inferred from the multiple alignments of the core genomes of the sampled strains using the HKY+G nucleotide substitution model and 1000 bootstrap replicates to assess statistical support for the tree internal branches, as previously described.^12^

**Correlation between the distance from root and the geographic distance of the sampling location of each strain from the epicenter of the epidemic.**
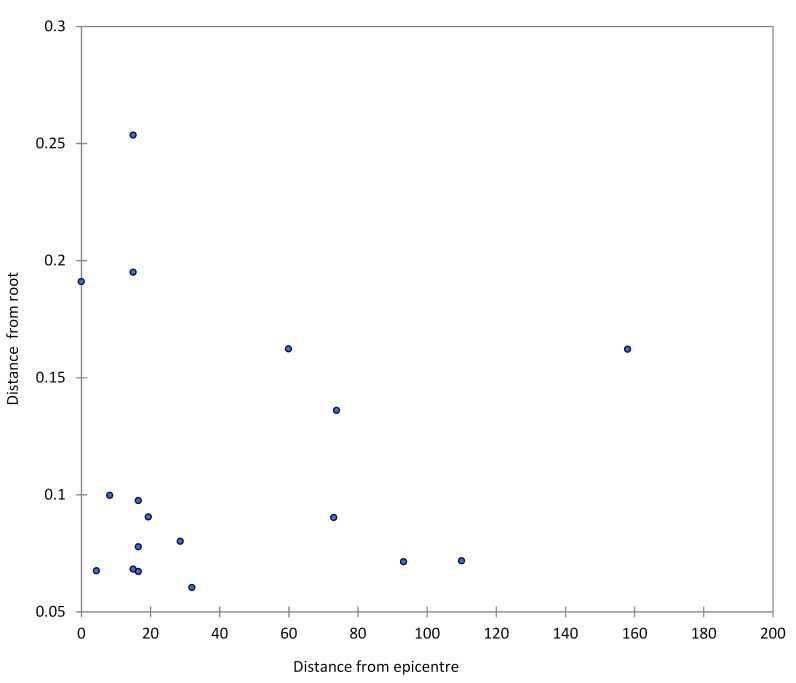
No correlation was found between genetic distances from root and geographic distance from epicenter, which may reflect the impact of human mobility.

The maximum likelihood phylogeny of the strains (Figure 2C) displays the typical star-like topology and intermixing of strains from different locations consistent with an exponential outbreak around a central area, resulting in the rapid spread of closely related strains. Indeed, no internal branch in the tree had significant bootstrap support (values were all consistently <60%), except for the branch clearly separating strain CMR001, one of the earlier strains collected in the epicenter of the outbreak (purple circle, Figure 2A), from all other strains (100% bootstraps). A potential limitation of this study is that the low diversity of the sample may diminish the power of phylogenetic inference, although it may in itself be an indication of exponential growth and the absence of repeated transmission from a more diverse environmental reservoir.

## Conclusions

This study provides data on whole genome sequence analysis of *V. cholerae* isolates from the South West Region-Cameroon. While the epidemic curve shows multiple peaks, there was no evidence of seasonality, nor has there been major recurrence of illness in subsequent years: i.e., we did not see the annual seasonal epidemics described in Asia and other regions of the world. Movement of strains through local environmental reservoirs might be expected to show correlation between genetic distance and geographic distance of sampling locations, with genetic heterogeneity generally increasing with progressively more distant locations from the initial epicenter of epidemic. Moreover, phylogenetic relationships among sampled strains would show highly supported monophyletic clades, corresponding to different transmission chains. On the contrary, the star-like topology of the tree and the lack of correlation between genetic and geographic distance seen with the South West Region outbreak are highly consistent with an exponential outbreak localized around a relatively restricted area (i.e., the city of Buea) with occasional spillover to other parts of the region likely mediated by occasional human-to-human contact. Intriguingly, one of the earliest sampled strains from the epicenter of the epidemic was significantly separated from all other strains, and appeared to be a natural outgroup for the tree; and thus, this phylogenetic analysis suggests this natural outgroup as the origin of the epidemic around the urban area (i.e., the city of Buea). In separate studies of spatial-temporal clustering of cholera cases in the Equatorial Monsoon^3^ climate subzone, we have found that cholera cases clustered in health districts with highways (RR = 3.99, CI95% 1.08-12.40) (Ngwa *et al.*, unpub. data), again supporting the idea of transmission through human movements. Taken together, the evidence is in support of the model that an index case probably migrated and triggered the outbreaks in the region as opposed to the ingestion of *V. cholerae* from an environmental reservoir. Antimicrobial resistance patterns mirror those reported from other areas of Cameroon during the 2010/11 epidemics;^7^^,^ 8 ^,^ 14 of note, resistance was not seen to any of the antibiotics used as “front line” agents against cholera, including tetracycline, ciprofloxacin, and azithromycin.

Over the past decades, sub-Sahara Africa has emerged as the primary contributor to the global cholera disease burden. From 2001-2009, 93% to 98% of all reported cases worldwide were from Africa, and in 2014, Africa was the leading global source of cholera burden, with 105,287 cases and 1,882 deaths reported from 19 countries.^15^ Some 20 years ago, Colwell proposed what has come to be known as the ‘cholera paradigm’,^16^ the concept that establishment of cholera endemicity requires the presence of aquatic environmental reservoirs, with seasonal/weather related increases in toxigenic *V. cholerae* O1 in these reservoirs serving as the primary trigger for human epidemics.^17^^,^ 18 In recent systematic reviews, Rebaudet *et al*. have challenged this conceptual framework, insisting that the ‘cholera paradigm’ does not apply to Africa.^4^^,^ 5 Instead, they hypothesize that cholera transmission in Africa is a function of human-to-human interactions, with human displacements (internal and external) serving as the major determinant of spread and trigger for recurrent epidemics. Our data, at least for the region under study in Cameroon, support this latter hypothesis. This, in turn, has implications for cholera management within Africa, underscoring the importance of monitoring (and limiting) movement of potentially infected persons into regions without active cholera epidemics.

## Competing Interests

The authors have declared that no competing interest exists.

## Supplements

**Figure:**
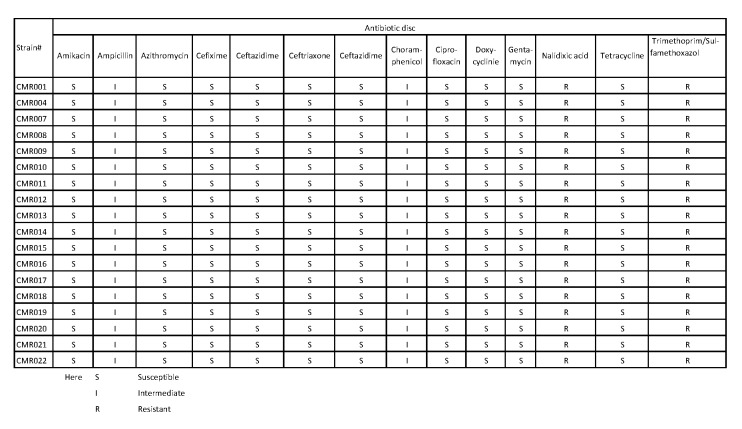
**Supplemental Table S1. Susceptibility results of V. cholerae strains from South West Region-Cameroon**

**Figure:**
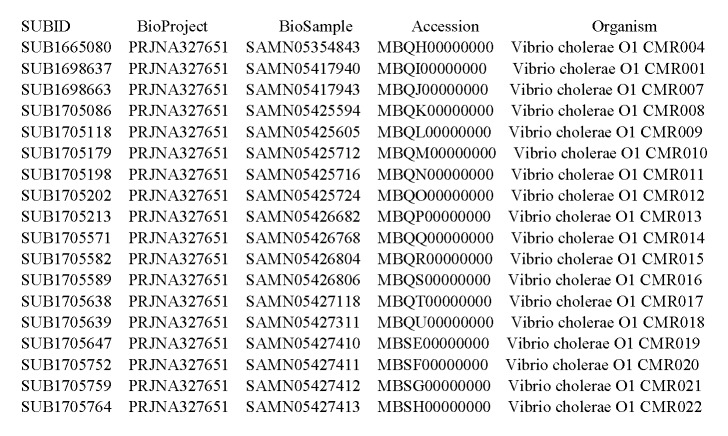
**Supplemental Table S2. Accession number of whole genome sequenced Isolates from South West Region, Cameroon cholera outbreak in 2010/2011**
